# Gut Microbiome-Host Metabolome Homeostasis upon Exposure to PFOS and GenX in Male Mice

**DOI:** 10.3390/toxics11030281

**Published:** 2023-03-19

**Authors:** Faizan Rashid, Veronika Dubinkina, Saeed Ahmad, Sergei Maslov, Joseph Maria Kumar Irudayaraj

**Affiliations:** 1 Biomedical Research Center, Mills Breast Cancer Institute, Carle Foundation Hospital, Urbana, IL 61801, USA; 2 Department of Comparative Biosciences, College of Veterinary Medicine, University of Illinois Urbana-Champaign, Urbana, IL 61801, USA; 3 Department of Bioengineering, University of Illinois Urbana-Champaign, Urbana, IL 61801, USA; 4 Carl R. Woese Institute for Genomic Biology, University of Illinois Urbana-Champaign, Urbana, IL 61801, USA; 5 Micro and Nanotechnology Laboratory, University of Illinois Urbana-Champaign, Urbana, IL 61801, USA; 6 Cancer Center at Illinois, University of Illinois Urbana-Champaign, Urbana, IL 61801, USA

**Keywords:** PFOS, GenX, microbiome, metabolome, small intestine, colon, gut microbiota–host metabolome homeostasis

## Abstract

Alterations of the normal gut microbiota can cause various human health concerns. Environmental chemicals are one of the drivers of such disturbances. The aim of our study was to examine the effects of exposure to perfluoroalkyl and polyfluoroalkyl substances (PFAS)—specifically, perfluorooctane sulfonate (PFOS) and 2,3,3,3-tetrafluoro-2-(heptafluoropropoxy) propanoic acid (GenX)—on the microbiome of the small intestine and colon, as well as on liver metabolism. Male CD-1 mice were exposed to PFOS and GenX in different concentrations and compared to controls. GenX and PFOS were found to have different effects on the bacterial community in both the small intestine and colon based on 16S rRNA profiles. High GenX doses predominantly led to increases in the abundance of *Clostridium sensu stricto*, *Alistipes*, and *Ruminococcus*, while PFOS generally altered *Lactobacillus*, *Limosilactobacillus*, *Parabacteroides*, *Staphylococcus*, and *Ligilactobacillus*. These treatments were associated with alterations in several important microbial metabolic pathways in both the small intestine and colon. Untargeted LC-MS/MS metabolomic analysis of the liver, small intestine, and colon yielded a set of compounds significantly altered by PFOS and GenX. In the liver, these metabolites were associated with the important host metabolic pathways implicated in the synthesis of lipids, steroidogenesis, and in the metabolism of amino acids, nitrogen, and bile acids. Collectively, our results suggest that PFOS and GenX exposure can cause major perturbations in the gastrointestinal tract, aggravating microbiome toxicity, hepatotoxicity, and metabolic disorders.

## 1. Introduction

In comparison to the human genome, the gut microbiota accounts for unique genes that number approximately 100 times those of the human genome [1]. These genes encode a variety of enzymes that help metabolize environmental chemicals driving a number of remarkable metabolic activities in the gastrointestinal microenvironment, some of which are crucial for host health [2,3,4]. Thus, disruption of the normal gut microbiota due to chemical toxicity can impact human health [5,6,7,8]. In addition, gut microbiota can significantly alter the host response to environmental pollutants, further highlighting the importance of the microbiome when determining the potential toxicity mechanisms of environmental chemicals [7].

These microbial–chemical interactions can be divided into toxicant modulation of the microbiome (TMM) and microbiome modulation of toxicity (MMT) [9]. TMM can include many effects, such as regulation of microbial genes and inhibition of specific enzymes, leading to alterations in the diversity and abundance of microbes in the community. MMT can alter the toxicity of a compound through the microbial enzymatic mechanisms of absorption, metabolism, disposition, and excretion [9,10]. These alterations affect enzyme families, mainly β-glucuronidases, azoreductases, and nitroreductases, that are involved in essential chemical processes in organisms [11,12,13]. In a recent database, around 529 gut microbes were reported to affect more than 1369 xenobiotic compounds through biocatalytic reactions [14]. Specific taxa have been associated with various health outcomes; e.g., susceptibility or resistance to toxic chemicals or drugs, the capability to recover from injury induced by a chemical agent, the presence of phenotypes associated with the disease, and the integrity of immune function [9].

Since the 1950s, perfluoroalkyl and polyfluoroalkyl substances (PFAS) have been some of the most commonly used compounds for both domestic and industrial purposes [15,16]. The most prominent chemical contaminants in this group of synthetic chemicals include perfluorooctanesulfonic acid (PFOS), 2,3,3,3-tetrafluoro-2-(heptafluoropropoxy) propanoic acid (GenX), and perfluorooctanoic acid (PFOA), which have been previously associated with various toxicities [17,18,19,20,21]. Among them, PFOS has been widely used as a surfactant [22] and reported to be present in human populations, leading to concern [23,24]. The half-life of PFOS in human serum is 3.4–5.4 years; thus, it has high potential to impact health [16,25,26], primarily by causing hypercholesterolemia, hyperuricemia, hypertension, hyperlipidemia, hyperglycemia, hepatotoxicity, neurotoxicity, and developmental toxicity [27,28,29,30,31,32].

GenX is a PFAS compound launched as an alternative to PFOA in 2009 and thought to be less toxic [33]. GenX has been found to have a shorter half-life of approximately 5 h [34]. Research in rats revealed a role for GenX in PPARα activation in fetal and maternal hepatic tissues. Further, reductions in the levels of lipids and thyroid hormones in the serum of the maternal rat were noted [35]. Recently, GenX was linked with hepatomegaly and developmental toxicity due to abnormal metabolism of glucose and amino acids, which may be associated with low birth weight and neonatal mortalities [36,37]. It is believed that the shorter-chain GenX is less deleterious to human health owing to its short half-life and low bioaccumulation [33]. Despite that, in vitro [18,38] and in vivo [35] studies indicate otherwise.

Considering the possible mechanisms through which the gut bacterial community can influence the toxicity and distribution of chemicals [5,6], it is intriguing to study gut microbiota changes in response to toxic chemical exposures. The association between various metabolic (mainly liver) disorders and PFOS and GenX exposure emphasizes the need to evaluate the effects of PFOS and GenX on gut microbiota and the associated metabolome. However, there is a significant gap in the literature on the impact of PFOS and GenX on different parts of the gastrointestinal system. In this study, we used a combination of analysis tools—i.e., 16S rRNA sequencing and untargeted metabolite profiling using LC-MS/MS analysis—to reveal the effect of PFOS and GenX exposure on the small intestine and colon microbiome and metabolome, as well as on the liver metabolome, in mice. We investigated the key changes induced by low, mild, and high dietary exposure to PFOS and GenX in the gut–liver axis to elucidate their potential role in the development of various host metabolic disorders. We detected noticeable perturbations in bacterial composition and metabolite profiles across the gut and liver, some of which were dose-dependent. A number of differential changes observed when comparing PFOS and GenX treatments suggested differences in their toxicity and metabolism. Our findings contribute to the understanding of the effects of PFOS and GenX on the gut microbiome and liver homeostasis.

## 2. Materials and Methods

### 2.1. Chemicals and Dosing Concentrations 

PFOS and GenX (97% purity) were purchased from Synquest laboratories, and Tween 20 was purchased from Sigma-Aldrich (St. Louis, MO, USA). PFOS and GenX stock solutions were prepared in 0.5% Tween-20 and deionized water. Three doses of both PFOS—i.e., 5, 10, and 20 mg/kg/day—and GenX—i.e., 10, 20, and 100 mg/kg/day—were created by diluting stock solutions. A vehicle control was prepared for the dosing of mice in which no PFOS or GenX was added. These dosing concentrations were selected based on previous studies in populations exposed to PFAS compounds through both the environment and occupation. The concentration of PFOS in the human body can reach up to 0.1 µg/g of body weight [39]. Studies with fish samples in the environment have noted concentrations of up to 9 µg/g [40]. According to the previous studies of GenX in mice (CD-1), the no observed adverse effect level (NOAEL) was found to be 5 mg/kg/day [41]. Another study in rats revealed abnormal LDL levels at 125 mg/kg/day and abnormal T3 hormone levels at 30 mg/kg/day doses [35]. Considering the results of the above studies, we selected three dosing concentrations for PFOS—i.e., 5 mg/kg, 10 mg/kg, and 20 mg/kg; three dosing concentrations for GenX—i.e., 10 mg/kg, 20 mg/kg, and 100 mg/kg; and two vehicle control groups for PFOS and GenX each.

### 2.2. Animals, Dosing, and Tissue Harvesting 

Adult CD-1 male mice were purchased from Charles River, USA. The animal experiments were conducted according to a protocol approved by the University of Illinois Urbana-Champaign (UIUC) Institutional Animal Care and Use Committee (IACUC). We chose to include only male animals to avoid possible variations due to diestrus cycles in females. A total of n = 44 male mice were randomly divided into eight groups (n = 6 biological replicates for PFOS and n = 5 for GenX in each group; three different concentration groups for each chemical and control group). Two to three animals were housed in ventilated polysulfone cages (changed once a week) at 25 °C with 12 h light and dark cycles. Mice were given water filtered via reverse osmosis in polysulfone bottles ad libitum and fed with Harlan Teklad Rodent Diet 8604. After 2 weeks of acclimatization for the PFOS group and 11 days for the GenX treatment group, the 60 day old mice were dosed orally once per day using pipette tips with either PFOS or GenX in various doses or the vehicle control dissolved in deionized water and Tween 20 for 14 consecutive days. Each animal was given a certain volume of the compound solution according to its weight (see Supplementary Table S1 for animal weights).

After dosing for 14 days, CO_2_ asphyxiation was used to euthanize the mice. Intestinal content was immediately collected for microbiome analysis in sterile Eppendorf tubes from the small intestine and colon regions after euthanasia under aseptic conditions. Similarly, liver organs were harvested for metabolome analysis and kept in sterile tubes. All tubes were snap-frozen in liquid nitrogen and stored in a −80 °C freezer until further use.

### 2.3. Microbial Profiling through 16S rRNA Sequencing 

#### 2.3.1. DNA Extraction, Library Preparation, and Sequencing

The DNA from the 200 mg gut microbiota samples of mice small intestine and colon were extracted separately from each mouse with a QIAamp PowerFecal Pro DNA kit (Qiagen, Hilden, Germany) per the manufacturer’s instructions. The DNA extracted from all samples was submitted to the DNA Services Facility at the Roy J. Carver Biotechnology Center (UIUC) for library preparation and sequencing per their standard protocol [42]. DNA used in PCR amplification was approximately 1 ng. PCR amplification of the V4 (515F–806R) region was accomplished with primers 5′-GTGYCAGCMGCCGCGGTAA and 5′-GGACTACNVGGGTWTCTAAT using the Fluidigm protocol (Fluidigm Corp., San Francisco, CA, USA). bcl2fastq 2.20 (Illumina, San Diego, CA, USA) was used to convert raw fastq files into demultiplexed compressed fastq files.

#### 2.3.2. Bioinformatic Analysis

We used trimmomatic v0.38 to remove 20 nt barcodes from the start of the reads. Raw sequences for the colon and small intestine were then processed using QIIME v2-2020.8 and the reads were denoised using DADA2 [43]. To avoid biases generated by differences in sequencing depth, the amplicon sequence variant (ASV) table was rarefied to 10,000 sequences per sample. ASV taxonomy was determined using the RDP v18 naive Bayes classifier with 80% confidence cut-off [44]. Ultimately, we estimated coverage for ASV characterization as 70 ± 17%. Due to the limitations of the resolution for taxonomical classification using the 16S gene sequencing technique, all analyses were restricted to the genus level. We used the Phylogenetic Investigation of Communities by Reconstruction of Unobserved States (PICRUSt) 2.3.0 pipeline to estimate the genomic repertoire of the detected ASVs to predict the functional capabilities of microbial communities [45].

### 2.4. Metabolite Profiling

#### Sample Preparation and LC-MS/MS Analysis

Liver tissues and fecal samples (from the small intestine and colon) were processed for metabolomic analysis. The wet weight of fecal samples was measured for use in normalization later. The samples were dissolved in 1 mL of the solvent—i.e., chloroform: methanol (2:1 *v*/*v*)—and homogenized using Omni Digital Tissue Homogenizer Thq. After homogenization, the final liquid, along with the homogenized samples inside, was delivered in O-ring-sealed, screwed vials in dry ice to the Metabolomics Facility at the Roy J. Carver Biotechnology Center (UIUC) for LC-MS/MS analysis. The analysis was performed by the Metabolomics Facility using their standard protocol [46]. 

Thermo Compound Discoverer v.3.2 was used for further analysis of the LC-MS/MS data to align the chromatogram and identify/quantitate the compounds. The Untargeted Metabolomics with Statistics to Detect Unknowns with ID Using Online Databases and mzlogic workflow was used. The Spectra settings used were: min. precursor mass = 0 Da, max. precursor mass = 5000 Da, and polarity mode = 1. The data were first analyzed in the (+) mode and then in the (−) mode. Align Retention Time settings were set as: max. Shift = 2 min and Mass tolerance = 5 ppm. Detect Compounds settings were set as: Intensity tolerance = 30%, Mass tolerance = 5 ppm, and minimum peak intensity = 1,000,000. Compound Discoverer software was used with four different data sources (mzCloud, Metabolika, ChemSpider, and Predicted Compositions) to assign compound annotations (chemical names and structures). In ChemSpider, five different databases (BioCyc [47], Human Metabolome Database (HMDB) [47], Kyoto Encyclopedia of Genes and Genomes (KEGG) database [48], Massbank, and the National Institute of Health (NIH) clinical collection) were used to search for and annotate the detected metabolites based on their molecular weight.

### 2.5. Statistical Analysis

Downstream analysis of taxonomic and metabolomic data was performed in R v4.0.2. ASV counts and transformations into relative abundance, Shannon diversity, and Bray–Curtis dissimilarity were calculated using the vegan package. We used the Bray–Curtis dissimilarity measure and principal coordinate analysis (PCoA) to visualize the differences in the microbial diversity between the various treatment groups. Linear regression analysis was used to test for associations between taxa and the concentrations of toxic compounds. Before testing for taxa differences, all taxa below 0.5% summary abundance across all samples were removed. 

For the metabolomic data, the peaks that had ∆Rt < 0.2 and ∆M < 0.01 were considered duplicates and were removed from further analysis. The peak areas were normalized according to the weights of samples. The area peak counts were log-transformed and z-score-normalized to stabilize the variance in the data. We used MetaboAnalyst v5.0 to perform statistical and enrichment analyses of the metabolomic data [49] and principal component analysis (PCA) was used to visualize the differences in the metabolic diversity between the various treatment groups and control groups. 

Prior to statistical analysis, 40% of the metabolites were filtered using the interquartile range (IQR) to remove metabolites with low variability. Identification of important metabolites separating the various treatments in the PFOS/GenX group was undertaken using one-way ANOVA with FDR correction or a linear regression test. Metabolites elevated/lowered in PFOS and GenX diets were selected as per the sign from the linear regression and the *p*-value of the fit. The set of elevated/lowered metabolites was used to perform pathway enrichment analysis against the KEGG [48] and Small Molecule Pathway (SMPDB) [50] databases. 

## 3. Results

**Figure 1:**
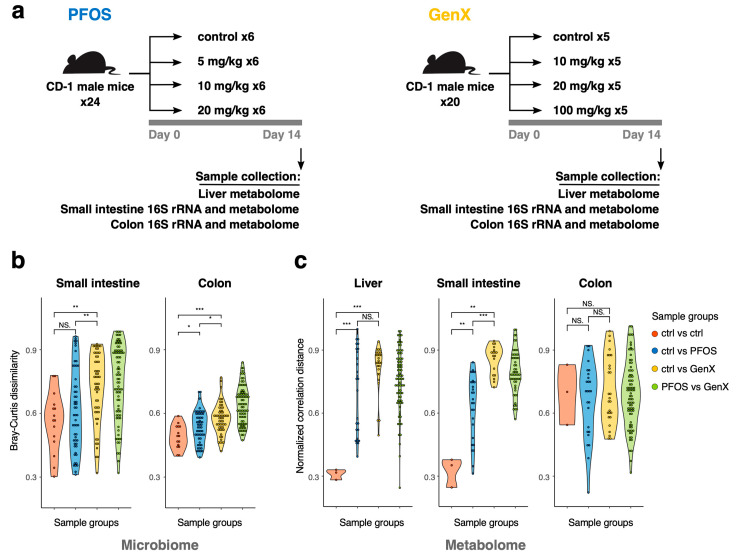
**General outline of experiments and sample diversity.** (**a**) Experimental setup. Mice were fed for two weeks with a diet containing either PFOS (in four different concentrations: control, 5 mg/kg, 10 mg/kg, and 20 mg/kg) or GenX (in four different concentrations: control, 10 mg/kg, 20 mg/kg, and 100 mg/kg). At the end of the experiments, animals were sacrificed and samples from the colon and small intestine microbiome and metabolome, as well as the liver metabolome, were collected. (**b**) Violin plots for Bray–Curtis dissimilarity for 16S rRNA samples from small intestine and colon. Red indicates sample similarity within the control group (n = 6), blue corresponds to similarity between the control group and PFOS-containing diets (n = 9), yellow—control and GenX (n = 9), and green—PFOS and GenX. Asterisks indicate the significance level of the two-sided Mann–Whitney test: *p* > 0.05 (NS), *p* < 0.05 (*), *p* < 0.01 (**), *p* < 0.001 (***). (**c**) Violin plots for normalized correlation distance between untargeted metabolome samples for the liver, small intestine, and colon with the same comparison groups as in (**b**).

The experimental design and an overview of the collected data are shown in Figure 1a. Adult male mice were subjected to a diet containing PFOS or GenX in three different concentrations, as well as a vehicle control (control, 5 mg/kg, 10 mg/kg, and 20 mg/kg for PFOS and control, 10 mg/kg, 20 mg/kg, and 100 mg/kg for GenX). After two weeks on a diet, samples were collected from three animal replicates for 16S rRNA sequencing of the mice colon and small intestine and untargeted LC-MS/MS metabolome profiling of liver tissue. The backup samples from the remaining replicates were kept in reserve to be used for untargeted metabolome profiling of the colon and small intestine microbiome.

Overall, we detected 818 representative ASVs that were assigned to 73 unique genera in the 16S rRNA samples (Tables S2 and S3). Totals of 5251, 3644, and 4392 unique metabolite features were detected in the colon, small intestine, and liver, respectively. Among them, 1185 (colon), 734 (small intestine), and 1098 (liver) had putative structures assigned (Tables S4–S6). We analyzed these multi-omics data to elucidate the impact of toxic compounds on different segments of the gastrointestinal tract. 

### 3.1. PFOS and GenX Exposure Impacts Microbial and Metabolome Diversity

The beta diversity analysis of both the colon and small intestine microbiomes revealed differential effects for the various concentrations of PFOS and GenX on the mice microbiomes (Figure S1a,b; PERMANOVA test for Bray–Curtis dissimilarity: small intestine: F = 1.67, R = 0.42, *p*-value < 0.05; colon: F = 1.60, R = 0.41, *p*-value < 0.01). Samples from the mice on GenX diets were separated from those exposed to PFOS (Figure S1a,b and Figure 1b—green group of dissimilarities). Moreover, GenX had a relatively stronger effect on the beta diversity compared to PFOS, with a relatively more significant effect in the small intestine in comparison to the colon (Figure 1b, blue vs. yellow groups of dissimilarities), as the corresponding samples were spread apart on the PCoA plot (Figure S1b), which may have signified taxonomic changes driven by its toxicity. There were no effects on the alpha diversity for either GenX or PFOS (*p*-value of linear regression for Shannon index > 0.05, Figure S1c), which is supported by a previous study on PFOS [51].

**Figure 2:**
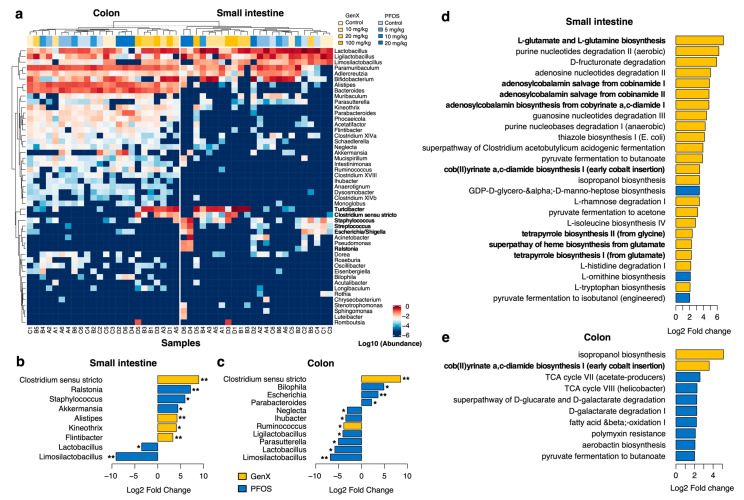
**PFOS and GenX metagenome composition analysis.** (**a**) Heatmap of taxonomic compositions of all 16S samples at the genus level. The columns correspond to the samples; compound concentration in the diet is denoted by the top color bar. The figure shows the taxa with total relative abundance ≥0.5% across all samples. Hierarchical clustering was performed using the Euclidean metric and complete linkage. (**b**,**c**) Genera significantly associated with increases in PFOS (blue) and GenX (yellow) concentrations in the small intestine (**b**) and colon (**c**). Genera with regression *p*-value < 0.05 are marked with (*), and genera that passed a more stringent cut-off (*p*-value < 0.01) are marked with (**). (**d**,**e**) Microbial metabolic pathway abundances significantly enriched in PFOS (blue) and GenX (yellow) diets (*p*-value of linear regression model < 0.01) in the small intestine (**d**) and colon (**c**). Only pathways with |Log2 Fold Change| > 2 are shown.

Initially, we created two separate control groups for PFOS and GenX diets with 0 mg/kg concentrations of the target compounds. We confirmed using taxonomic analyses that these samples were similar to each other (Kolmogorov–Smirnov *p*-value < 0.01 for comparisons of Bray–Curtis dissimilarities within the control group and for the control group versus others). Thus, in the subsequent metabolomic analyses, we decided to use one group of control samples (marked as PFOS control in Figure S1a,b and Figure 2a).

Similar patterns of sample variation were observed for untargeted LC-MS/MS metabolome features (see Figure S1d–f; PERMANOVA test for normalized correlation distance: liver F= 1.64, R = 0.41, *p*-value < 0.05; small intestine: F = 2.07, R = 0.53, *p*-value < 0.05; colon: F = 2.08, R = 0.47, *p*-value < 0.05). There was a much more pronounced effect from the toxicity of the compounds and clearer separation of GenX and PFOS samples for the liver and small intestine compared to microbiome features (Figure 1b,c). Metabolome characteristics also resulted in a more consistent triplicate grouping than taxonomic features (Figure 1c and Figure S1d–f). Interestingly, the colon metabolome was much less affected by toxic compounds than the small intestine, while the effect on the colon microbiome was still significant (Figure 1b). 

### 3.2. PFOS and GenX Ingestion Causes Gut Microbial Alterations

Changes in taxonomic abundance at the phylum, family, and genus levels were observed in microbiota collected from both small intestines and colons exposed to PFOS and GenX. At the family level, we observed that families with high prevalence of pathogens such as *Enterobacteriaceae*, *Streptococcaceae*, and *Pseudomonadaceae* were associated with higher toxicity (Figure S2). The variation patterns observed in the PCoA plots (Figure S1a,b) were also evident in a heatmap of taxonomic compositions (Figure 2a). In general, replicates tended to cluster together, and samples treated with high concentrations of GenX tended to cluster separately from the PFOS-exposed samples. This separation was mostly driven by the increase in the abundances of the following genera: *Turicibacter*, *Streptococcus*, *Staphylococcus*, and *Clostridium sensu stricto*. We further assessed whether any of these genera were significantly associated with increases in GenX and PFOS concentrations (Figure 2b,c and Figures S3–S6).

With an increasing concentration of PFOS in the small intestine, we observed a significant increase in the abundance of the genus *Ralstonia*, and a significant reduction for the abundance of *Limosilactobacillus* (*p*-value of linear regression model < 0.01, Figure 2b). The abundances of several other bacterial genera were also found to vary (e.g., increase for *Staphylococcus* and *Akkermansia* and decrease for *Lactobacillus*) with increasing concentrations of PFOS (*p*-value of linear regression model < 0.05). In the colon, PFOS was found to significantly elevate the abundance of the genus *Escherichia/Shigella* and reduce that of *Limosilactobacillus* (*p*-value of linear regression model < 0.01, Figure 2c). Further, the abundances of several other bacteria increased (i.e., *Bilophila* and *Parabacteroides*) and decreased (i.e., *Neglecta*, *Ligilactobacillus*, *Ihubacter*, *Parasutterella*, and *Lactobacillus*) (*p*-value of linear regression model < 0.05). 

Similarly, with the increasing concentration of GenX in the small intestine, we noticed an increase in the abundances of *Clostridium sensu stricto*, *Alistipes*, and *Flinibacter* (*p*-value of linear regression model < 0.01, Figure 2b). There was also a qualitative increase in the abundance of *Kineothrix*. With increasing concentrations of GenX in the colon, the abundance of *Clostridium sensu stricto* increased and that of *Limosilactobacillus* decreased significantly (*p*-value of linear regression model < 0.01, Figure 2c). In addition to the alterations in the levels of the abovementioned bacteria, the abundances of several other bacteria were altered qualitatively in both the small intestine and colon in specific treatment groups. 

At higher concentrations of PFOS, slight decreases in Firmicutes abundance in the small intestine and colon, as well as a slight increase in Bacteroidetes abundance in the small intestine, were found in mice exposed to 5 and 10 mg/kg of PFOS. However, abundances of Bacteroidetes and Firmicutes in the small intestine and colon of GenX-exposed mice and the B/F ratio remained relatively constant (Figure S7).

### 3.3. Microbial Metabolic Pathways Are Altered in PFOS- and GenX-Exposed Mice 

We used PICRUSt reconstruction of the functional repertoire in 16S rRNA samples to identify pathways potentially enriched with PFOS and GenX diets (Figure 2d,e and Figures S10 and S11). In the small intestine, we detected 60 pathways that were significantly elevated and 5 that were significantly lowered with increases in the concentration of GenX (*p*-value of linear regression model < 0.01, fold change > 2). These included glutamate and glutamine biosynthesis, purine nucleotide degradation, tetrapyrrole and cobalamin (Vitamin B12) biosynthesis, and the super-pathways of BCAA and AAA biosynthesis (see Figure 2d). In contrast, only 21 microbial pathways were elevated with the PFOS diet (*p*-value of linear regression model < 0.01, fold change > 2), most of them being amino acid biosynthesis pathways (Figure S10): aromatic amino acid (AAA) biosynthesis, branched-chain amino acid (BCAA) biosynthesis, and pathways involved in the biosynthesis of L-arginine and L-lysine.

In contrast to the small intestine, in the colon, the majority of enriched/lowered pathways were associated with the PFOS diet (see Figure 2e). We found 15 significantly elevated and 5 significantly lowered pathways (*p*-value of linear regression model < 0.01, fold change > 2) for PFOS and only 4 significantly elevated ones for GenX (Figure S11). In the colon, PFOS enriched several microbial pathways, mainly those involved in pyruvate fermentation for butanoate and acetone, L-lysine fermentation for butanoate and acetate, TCA cycle VII (acetate producers), the super-pathways of D-glucarate and D-galactarate degradation, and the pathways of polymyxin resistance. In general, pathways enriched with the PFOS diet were related to various fermentation and oxidation reactions and could reflect the level of oxidative stress of this compound.

### 3.4. PFOS and GenX Ingestion Alters the Small Intestine Metabolome in Mice

We identified 383 (11%) metabolite variables that significantly differed between groups (FDR-adjusted one-way ANOVA *p*-value < 0.01) in the small intestine metabolome. Among the most distinctive (FDR-adjusted one-way ANOVA *p*-value < 0.0001), 38 had structures assigned to them (Figure S8). There were two main clusters of metabolites: metabolites highly elevated with GenX-containing diets (Figure S8d) and metabolites that were underrepresented in GenX samples (Figure S8e). There was also a small set of metabolites that were characteristic of the PFOS diet: panobinostat, norethisterone enanthate, 3-oxocholic acid, and quinbolone. Based on the linear regression analysis, we identified 73 lowered and 29 elevated metabolites (*p*-value of linear regression model < 0.05) for PFOS diets (Table S7) and 77 lowered metabolites and 1 elevated metabolite (*p*-value of linear regression model < 0.05) for GenX diets (Table S9). PFOS exposure resulted in alterations to several metabolites (e.g., cortisol, histidine, methyl testosterone, 2′-deoxyadenosine, protoporphyrin IX, etc.), along with changes in microbiota that were associated with disturbances in various important bacterial metabolic pathways, such as steroidogenesis, purine metabolism, and porphyrin metabolism (Figure S8, Table S7). GenX exposure affected the levels of other microbial metabolites (lyso PE, AAAs, citric acid, serine, theophylline, 2′-deoxycytidine, histidine, etc.) linked to changes in such bacterial metabolic pathways as phospholipid biosynthesis, the urea cycle, the citric acid cycle, AAA metabolism, oxidation of branched-chain and long-chain fatty acids, serine metabolism, purine metabolism, and pyrimidine metabolism (Figure S8, Table S9). 

### 3.5. PFOS and GenX Ingestion Alters Mice Colon Metabolome

We identified 137 (3%) metabolite variables that significantly differed between groups (FDR-adjusted one-way ANOVA *p*-value < 0.01) in the colon metabolome, indicating that lower parts of the digestive system are likely to be shielded from PFOS and GenX toxicity (Figure S9). Using linear regression analyses, we identified 33 lowered and 77 elevated metabolites (*p*-value of linear regression model < 0.05) for PFOS diets and 109 lowered and 22 elevated metabolites (*p*-value of linear regression model < 0.05) for GenX diets (Tables S7 and S9). Among the metabolites elevated in the PFOS group, we found several glucocorticoid-like molecules, such as cortisone, 11-ketoprogesterone, 3,17-dihydroxy-16-methyl-5,6-epoxypregnan-20-one, etc. Lowered metabolites included lyso PE, AAAs, citric acid, serine, theophylline, 2′-deoxycytidine, and histidine, and they led to disturbances in the metabolism of porphyrin, sphingolipid, arachidonic acid, and linolenic acid (Figure S9, Table S8). 

In the colon, GenX affected the levels of phytosphingosine, citric acid, arachidonic acid, AAAs, BCAs, and guanine. These metabolites are linked to the metabolism of sphingolipid, arachidonic acid, linolenic acid, tyrosine, purine, and phenylalanine, as well as the citric acid cycle (Figure S9, Table S10).

There was also a set of 25 metabolites that were affected with both PFOS and GenX diets (Figure S9c); however, almost half of them showed different effects between the diets (note that the metabolites had a positive regression slope for PFOS diets and negative for GenX).

### 3.6. PFOS and GenX Ingestion Alters Liver Metabolome

**Figure 3:**
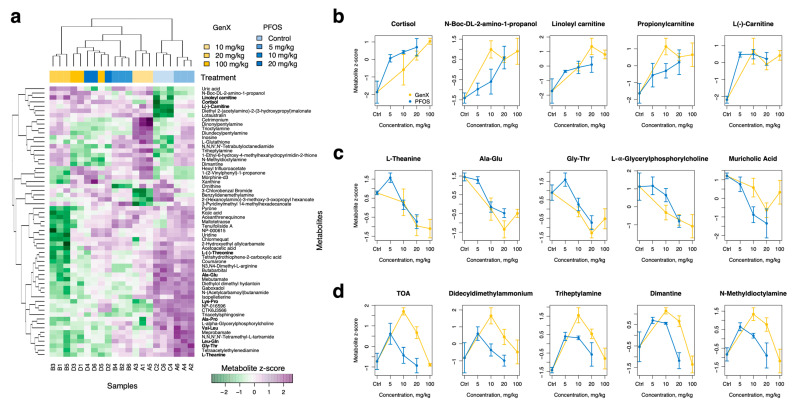
**Liver metabolites significantly varied with different diets.** (**a**) Heatmap of metabolites selected with one-way ANOVA test (*p*-value < 0.0001); peaks with low interquartile variability were filtered out before this analysis. Only metabolites with assigned potential chemical structures are shown. Colors represent z-score values of log10-normalized metabolite concentrations. The hierarchical clustering for samples and metabolites was performed using the Euclidean metric and complete linkage. (**b**) Metabolites that systematically increased with increases in the concentration of toxic compounds. (**c**) Metabolites that systematically decreased with increases in the concentration of toxic compounds. (**d**) Metabolites with nonlinear responses to the concentration of toxic compounds.

Untargeted metabolomic analysis using LC-MS/MS was undertaken to observe the general metabolomic alterations due to PFOS and GenX exposure. We used one-way ANOVA to identify metabolite features that differed between groups of replicates and noted 491 metabolites (11%) that were significant (FDR-adjusted *p*-value < 0.01) (Figure S12). Further, a subset of the most distinctive variables (*p*-value < 10^−4^) for which we were able to assign chemical structures (61 metabolites in total) were selected, as shown in Figure 3a. We further classified metabolites into three groups: metabolites that systematically increased with the concentration of toxic compounds (see Figure 3b for examples), metabolites that systemically decreased (Figure 3c), and metabolites that showed nonlinear responses to the concentration of toxic compounds (Figure 3d). The first group predominantly contained cortisol- and carnitine-containing compounds, which increased with increasing concentrations of PFOS and GenX. The second group consisted of various amino acids and dipeptides, such as L-theanine, L-(-)-threonine, alanine-glutamate, and glycine-threonine. There was also a cluster of amine-containing compounds that were elevated with intermediate concentrations of toxic compounds (Figure 3d).

We further analyzed the liver metabolome for PFOS- and GenX-containing diets separately to identify metabolites that were specifically enriched/decreased with increasing concentrations of a particular compound (Tables S10 and S11). 

**Figure 4:**
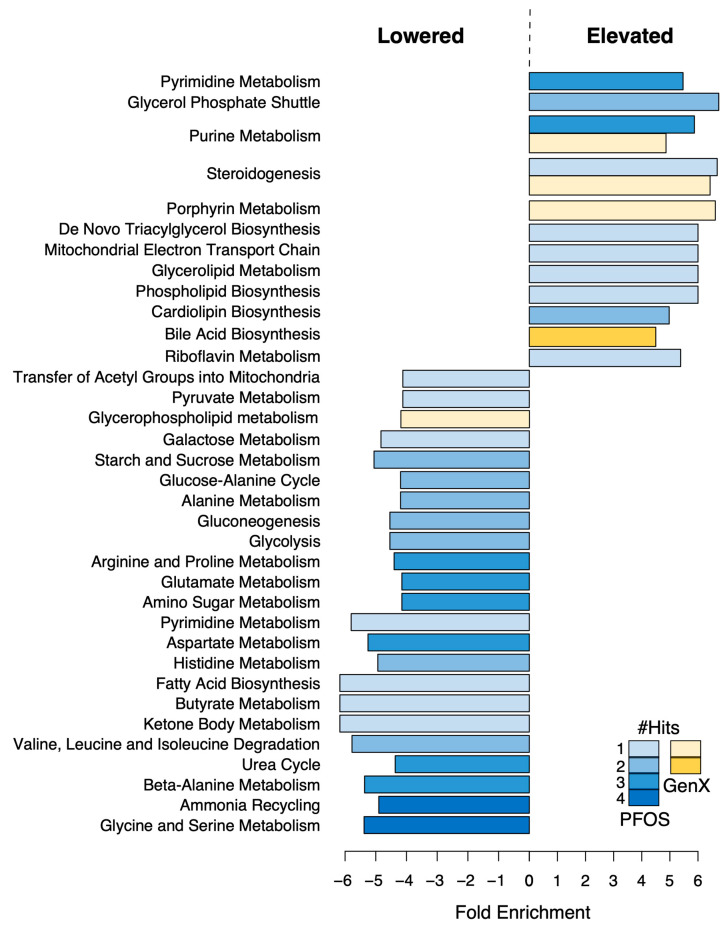
**Upregulated and downregulated pathways based on metabolite enrichment analysis in mice livers.** Only metabolites that showed a significant association with the concentration of the toxic compounds (*p*-value of linear regression model < 0.05) were used for this analysis. Blue bars indicate pathways enriched with the PFOS diet and yellow denotes GenX. The color intensity indicates the number of metabolites present in the enriched/lowered metabolite set that belonged to a given pathway. Pathway fold enrichment was calculated using QEA analysis. Only those with a non-zero influence score or multiple hits and FDR < 0.05 are shown. The negative-fold enrichment values indicate a set of pathways containing downregulated metabolites, while positive values indicate elevated ones.

For PFOS, we identified 147 underrepresented metabolites and 61 overrepresented ones (*p*-value of linear regression model < 0.05). Among the overrepresented metabolites were various fluorine-containing compounds (e.g., (2,3-difluorophenyl)acetic acid, 2,2,2-trifluoro-N-pentylacetamide, 2,6-difluoro-N-(tetrahydro-2-furanylmethyl)benzamide, hexyl trifluoroacetate, 9-hydrazono-2,7-bis-[2-(diethylamino)-ethoxy]-fluorene, etc.), which were probably products of PFOS degradation. The rest of the enriched metabolites were mostly organic acids, nucleic acids, fatty acyls, and carbohydrates, which were associated with lipid biosynthesis (glycerolipids, cardiolipins, phospholipid biosynthesis, etc.) and steroidogenesis pathways in the liver (Figure 4 and Table S8). In contrast, the underrepresented group primarily consisted of various amides and amino-acid compounds. A further reduced prostaglandin F2 level was found with an increase in PFOS concentration.

For GenX, we identified only 18 underrepresented compounds and 49 overrepresented ones (*p*-value of linear regression model < 0.05), as it is considerably less toxic and has a milder effect on the liver (Table S7). Most of the compounds that decreased in concentration with increases in GenX were organic acids and fatty acyls, as well as amine-containing compounds. For PFOS, among the enriched metabolites, we further detected fluorine-containing compounds (e.g., 9-hydrazono-2,7-bis-[2-(diethylamino)-ethoxy]-fluorene), various organic acids, fatty acyls, and benzenoids and sterol lipids, which are associated with lipid metabolism (see Figure 4). Further, the presence of high concentrations of GenX was associated with increased levels of protoporphyrine and bile acids (taurochenodesoxycholic acid; taurocholic acid; and lithocholic acid, 2TMS derivative) in the liver.

## 4. Discussion

In this study, we determined that PFOS and GenX exposure leads to significant perturbations in the gut microbiota and liver metabolome. Our results support previous studies demonstrating the roles of PFOS and GenX in metabolic disorders, endocrine toxicity, hepatotoxicity, developmental toxicity, altered metabolism, and several other kinds of toxicity [18,27,28,29,30,31,35,36,37]. We found that GenX had a relatively greater effect on the microbial beta diversity compared to PFOS and a much greater effect on the microbial diversity of the small intestine compared to colon (Figure 1b,c). 

Variation patterns similar to the gut microbiome were also observed in the metabolome features. Interestingly, colon and liver samples treated with higher concentrations of both PFOS and GenX had similar effects, while, for the small intestine, we observed different effects for GenX and PFOS. This may suggest that the colon and liver metabolome reflect a general toxicity level, while the small intestine is probably sensitive to a particular compound.

Different taxa were found to be affected by PFOS and GenX, with PFOS affecting more species than GenX in both parts of the gut. We further noted that fold changes in relative abundance were much higher for PFOS than for GenX in the range of concentrations studied. In general, exposure to toxic compounds suppressed populations of beneficial taxa, such as *Lactobacillus*, *Limosilactobacillus*, and *Ligilactobacillus*, and led to increases in opportunistic pathogenic clades, such as *Streptococcus* and *Turicibacter*. These findings are corroborated by recently published studies that have also revealed the direct effect of PFOS (after 17 and 21 days of exposure) on the cecal content of the mice gut microbiome [10,39,51]. However, there are several discrepancies between the findings (e.g., these studies reported an increase in *Clostridium cluster XIVa*, while our results showed a reduction in its levels for both chemicals; the previous studies reported depletion of *Alistipes*, while we observed a qualitative increase in the PFOS treatment group), which might have been due to the collection of gut microbiota from different parts of the intestine in these two studies. 

The pathway analysis of the gut microbiome revealed that, in the small intestinal community, both PFOS and GenX enriched microbial pathways involved in amino acid biosynthesis; specifically, AAA, BCAA, arginine, and lysine (Figure 2d). Elevated levels of AAA and BCAAs have been reported to be linked to metabolic liver disease (MLD), increased risk of obesity [52], insulin resistance, type 2 diabetes mellitus (T2D) [53], and heart failure [54]. Further, changes in the metabolism of arginine and lysine in the intestinal microbiota were found to disturb host physiology [55,56], while increases in arginine metabolism were linked to coronary heart disease [57]. In addition, for GenX exposure, there were many more enriched pathways, including glutamate and glutamine biosynthesis, purine nucleotide degradation, and cobalamin (Vitamin B12) and tetrapyrrole biosynthesis. Amyotrophic lateral sclerosis (ALS) and Alzheimer’s disease have been linked with perturbed levels of glutamine and glutamate [58,59], suggesting a possible association between GenX exposure and these diseases. Similarly, the enrichment of purine degradation pathways can alter the degradation of purine into uric acid (hyperuricemia), which may influence the development of gout [60,61]. Cobalamin is known to be involved in antioxidative stress response [62], as is glutamine [63], and affects the survival of gut bacteria during severe oxidative stress [62]. Therefore, GenX may have roles in several diseases through the modulation of microbial pathways. Moreover, there is some evidence that various tetrapyrroles, including B12, can be involved in PFAS degradation [64,65], highlighting the intriguing possibility that microbiome modulation due to GenX toxicity occurs as a response to environmental pollutants. This emphasizes the need for further mechanistic studies to provide insight into the mechanisms through which microbiome perturbations resulting from these chemicals may contribute to metabolic health disorders.

In contrast to the small intestine, in the colon, most enriched/lowered pathways were associated with the PFOS diet (Figure 2e). Elevated levels of acetate are associated with obesity since acetate participates as a substrate in adipocyte and hepatic lipogenesis [66], as well as promoting secretion of ghrelin and insulin, leading to an increase in fat storage [67]. Secondly, D-glucarate has been found to inhibit various cancers, and its degradation may lead to an increased risk of developing several cancers [68,69]. Further, the enrichment of the polymyxin resistance pathway in gut microbiota may affect antibiotic resistance against polymyxin in bacterial infections due to the presence of transferable genetic elements in the microbiota contributing to polymyxin resistance [70].

In agreement with the fact that a few taxa were altered in colon microbiota in GenX-treated mice, our analysis demonstrated enrichment of very few pathways (Figure S10). The most important ones included cob(II)urinate a c-diamide biosynthesis—i.e., an anaerobic pathway for the synthesis of vitamin B12 [71]—and the super-pathway of thiamin diphosphate (vitamin B1) biosynthesis. Vitamin B1 is involved in nucleic acid ribose metabolism, which may enhance resistance to chemotherapy and tumor survival, thus suggesting a possible role for GenX in promoting the tendency of tumors to grow and spread in the body [72].

PFOS has been found to alter the host metabolism in previous studies [10,51] and various metabolic disorders are associated with exposure to PFAS compounds [35,73]. Therefore, we performed untargeted metabolomic analysis using LC-MS/MS to observe the general metabolomic alterations resulting from PFOS and GenX dietary exposure. Cortisol- and carnitine-containing compounds were found to be increased in the liver metabolome upon exposure to PFOS and GenX. These metabolites are well-known for causing oxidative stress in host species [74,75], and the findings may suggest possible roles for PFOS and GenX in the causation of oxidative stress. The levels of various amino acids and dipeptides, such as L-theanine, L-(-)-threonine, alanine-glutamate, and glycine-threonine, were also reduced as PFOS and GenX concentrations increased, possibly indicating that amino acid biosynthesis pathways were disrupted, as shown in Figure 4. PFAS compound exposure has previously been linked to the disruption of amino acid metabolism, which supports our findings [76]. 

For PFOS, the other metabolites found to be enriched were mostly organic acids, nucleic acids, fatty acyls, and carbohydrates associated with lipid biosynthesis (glycerolipids, cardiolipins, phospholipid biosynthesis, etc.) and steroidogenesis pathways in the liver (Figure 4 and Table S8). This might suggest a possible mechanistic role for liver metabolite disruption due to PFOS exposure in non-alcoholic fatty liver disease [77]. In contrast, various amides and amino-acid compounds were downregulated, which led to downregulation of multiple pathways, including glycine, serine, alanine, valine, leucine, histidine, and aspartate metabolism; ammonia recycling; and the urea cycle, thus indicating that PFOS induced inhibition of nitrogen metabolism in mice livers (Figure 4). We further detected that the prostaglandin F2 level was reduced with increases in PFOS concentration, and decreased levels of prostaglandin F2 might be associated with severe disruption of endocrine regulation, along with elevated cortisol levels [78], thus suggesting a possible role for PFOS in endocrine disruption.

For GenX, limited numbers of compounds were found to be altered compared to PFOS, since GenX is considerably less toxic and has a milder effect on the liver (Table S7). The downregulated compounds were mainly organic acids and fatty acyls, as well as amine-containing compounds. Protoporphyrine was found to be elevated with increasing concentrations of GenX, which might have been associated with the degradation of GenX [64] and protection of the liver from the potential toxic damage posed by GenX. This finding was in agreement with the enriched biosynthetic pathways in the small intestine bacteria. Interestingly, unlike PFOS, the presence of high concentrations of GenX was associated with increased bile acid levels in the liver (taurochenodesoxycholic acid; taurocholic acid; and lithocholic acid, 2TMS derivative). This could potentially explain why microbial communities in the small intestine were so different in diets containing GenX compared to PFOS (Figure 1b,c), as bile acids can modulate bacterial communities in the upper intestine [79].

Overall, our findings demonstrate a differential, dose-dependent effect for PFOS and GenX on the gut microbiota and gut microbial metabolite secretion leading to alterations in the abundances of specific bacteria and metabolites. These changes may be linked to liver metabolome disruption through the gut–liver axis, which needs to be experimentally evaluated. Further, PFOS was found to have relatively greater impact on colon bacteria than GenX, perhaps due to the high potency of PFOS.

## 5. Conclusions

**Figure 5:**
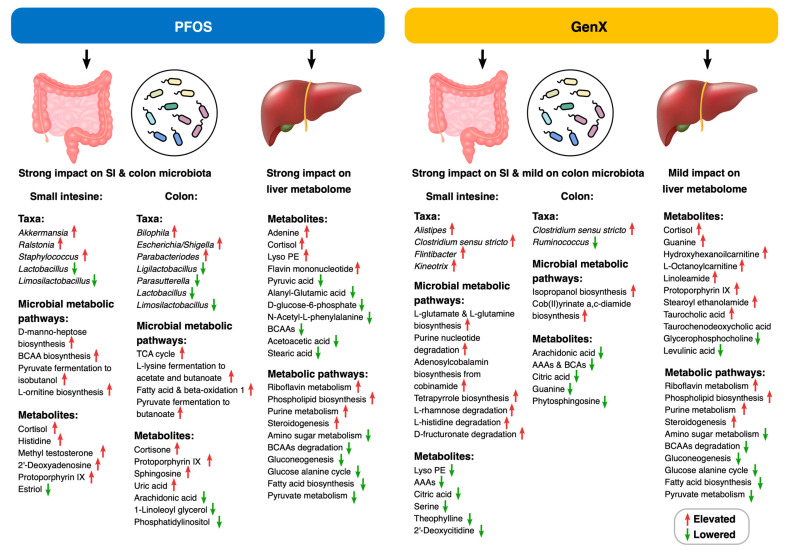
**Summary of gut microbiome and liver metabolome changes due to PFOS and GenX exposure.** Important small intestine and colon microbial (genera, microbial metabolic pathways, microbial metabolites) and liver (metabolites and metabolic pathways) features were found to be altered due to PFOS and GenX sub-acute exposure.

In the present study, PFOS and GenX exposure was found to alter the gut microbiota and potentially disrupt the microbial functional pathways and the liver metabolome, as delineated in Figure 5. Our findings indicated that PFOS exhibited a stronger impact on colon microbiota and liver metabolome compared to GenX. Moreover, exposure to toxic compounds suppressed the beneficial taxa populations and increased the abundances of opportunistic pathogenic clades. While the specific alterations were somewhat different for these compounds, our experiments indicated that both PFOS and GenX can affect several host–microbiome pathways involved in many essential metabolic pathways, among which steroidogenesis and metabolism (of carbohydrates, lipids, amino acids, bile acids, purines, and pyrimidines) are of high clinical significance. The direct impact of these compounds on the gut bacterial community and the mechanistic associations between these microbiome perturbations and liver metabolome changes suggest that their toxicity for liver metabolism could perhaps be regulated by the gut microbiome. Hence, both PFOS and GenX can significantly compromise host–microbiome homeostasis, resulting in metabolic disturbances in the host.

While the number of biological replicates in our study was limited, we used a combination of analysis tools and multi-omics data to ascertain the effects of toxicity on both the microbial community and the host. Evaluating several concentrations allowed us to strengthen the power of our analysis beyond qualitative determinations of significant changes across the gastrointestinal system. However, further studies with more replicates are needed to reinforce the results and distinguish real effects from natural sample variation. Studies with gnotobiotic animals evaluating changes in the effects of specific microbes and microbial metabolite alterations on host metabolome, transcriptome, and physiology due to PFAS exposure might provide exciting insights into the potency of these effects. Further, research on the impact of chronic PFAS exposure on gut microbiota and host metabolic homeostasis would be valuable in determining the impacts of these compounds on organisms exposed for prolonged duration.

## Data Availability

16S rRNA sequences are available via SRA with BioProject number PRJNA733227. Raw metabolomic spectra can be made available upon request to the authors. Tables of processed metabolite and ASV counts are available as Supplementary Tables S2–S6. The code for the analysis is available here: https://github.com/Zireae1/pfos_genx (accessed on 10 January 2023).

## Supplementary Materials

The following supporting information can be downloaded at: https://www.mdpi.com/article/10.3390/toxics11030281/s1, Figure S1: Beta-diversity plots for multi-omics data collected in the study; Figure S2: Heatmap of the taxonomic compositions of all 16S samples at the family level (according to rdp classification); Figure S3: Changes in genus levels for 16S samples for small intestine and PFOS diets; Figure S4: Changes in genus levels for 16S samples for small intestine and GenX diets; Figure S5: Changes in genus levels for 16S samples for colon and PFOS diets; Figure S6: Changes in genus levels for 16S samples for colon and GenX diets; Figure S7: Changes in phylum levels for 16S samples for the small intestine (a) and colon (b) (>80% confidence in rdp classification); Figure S8: Small intestine metabolites significantly varied between different diets; Figure S9: Colon metabolites significantly varied between different diets; Figure S10: Differentially abundant pathways in the small intestine microbiome; Figure S11: Differentially abundant pathways in the colon microbiome; Figure S12: Heatmap of liver metabolites that significantly varied between different diets; Table S1: Dosing concentration and weight for each animal; Table S2: Relative abundances of microbial genera in the small intestine samples; Table S3: Relative abundances of microbial genera in the colon samples; Table S4: Metabolite raw peak counts in small intestine samples; Table S5: Metabolite raw peak counts in colon samples; Table S6: Metabolite raw peak counts in liver samples; Table S7: Small intestine metabolites significantly enriched/lowered with PFOS diet; Table S8: Colon metabolites significantly enriched/lowered with PFOS diet; Table S9: Small intestine metabolites significantly enriched/lowered with GenX diet; Table S10: Colon metabolites significantly enriched/lowered with GenX diet; Table S11: Liver metabolites significantly enriched/lowered with PFOS diet. Table S12: Liver metabolites significantly enriched/lowered with GenX diet.
