# Supplementary material for: Gut Microbiome-Host Metabolome Homeostasis upon Exposure to PFOS and GenX in Male Mice
Source: Toxics. 2023 Mar 19;11(3):281. doi: 10.3390/toxics11030281 (PMC10051855; doi:10.3390/toxics11030281)
Supplement: Supplementary file 1 [file toxics-11-00281-s001.zip › toxics-2217555-Supplementary--Figures.pdf]

Supplementary

Gut Microbiome-Host Metabolome Homeostasis upon Exposure to PFOS and GenX in Male Mice

Faizan Rashid [1,2,†], Veronika Dubinkina [3,4,†], Saeed Ahmad [1,3], Sergei Maslov [3,4] and Joseph Maria Kumar Irudayaraj [1,2,3,5,6,*]

1 Biomedical Research Center, Mills Breast Cancer Institute, Carle Foundation Hospital, Urbana, IL 61801, USA
2 Department of Comparative Biosciences, College of Veterinary Medicine, University of Illinois Urbana-Champaign, Urbana, IL 61801, USA
3 Department of Bioengineering, University of Illinois Urbana-Champaign, Urbana, IL 61801, USA
4 Carl R. Woese Institute for Genomic Biology, University of Illinois Urbana-Champaign, Urbana, IL 61801, USA
5 Micro and Nanotechnology Laboratory, University of Illinois Urbana-Champaign, Urbana, IL 61801, USA
6 Cancer Center at Illinois, University of Illinois Urbana-Champaign, Urbana, IL 61801, USA
* Correspondence: jirudaya@illinois.edu; Tel.: +1-217-300-0525
† These authors contributed equally to this work.

Supplementary Figures

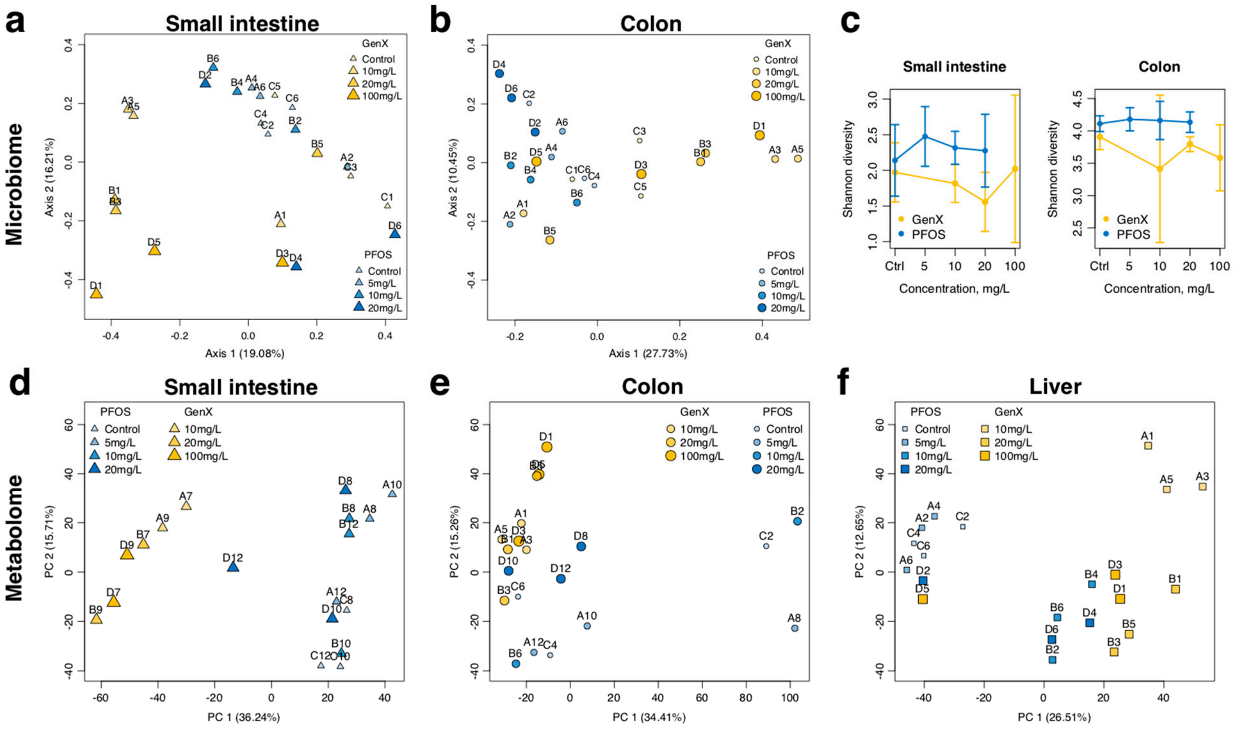

Figure S1. Beta-diversity plots for multi-comics data collected in the study. (a-b) Principal coordinates analysis (PCoA) based on Bray-Curtis dissimilarity of 16S rRNA samples. PCoA for small intestine (a) and colon (b). Blue points correspond to PFOS, yellow points to GenX, circles and triangles represent colon and small intestine respectively. The size and color intensity of the points correspond to the concentration of chemicals in the diet. We encoded animals as A1, A2, etc., and indicated respective samples on each plot. (c) Shannon diversity index for 16S rRNA data collected for different compound concentrations. (d-f) Principal component analysis (PCA) for all metabolomics variables. (d) small intestine, (e) colon and (f) for liver. Percentages on axes show the proportion of variance explained.

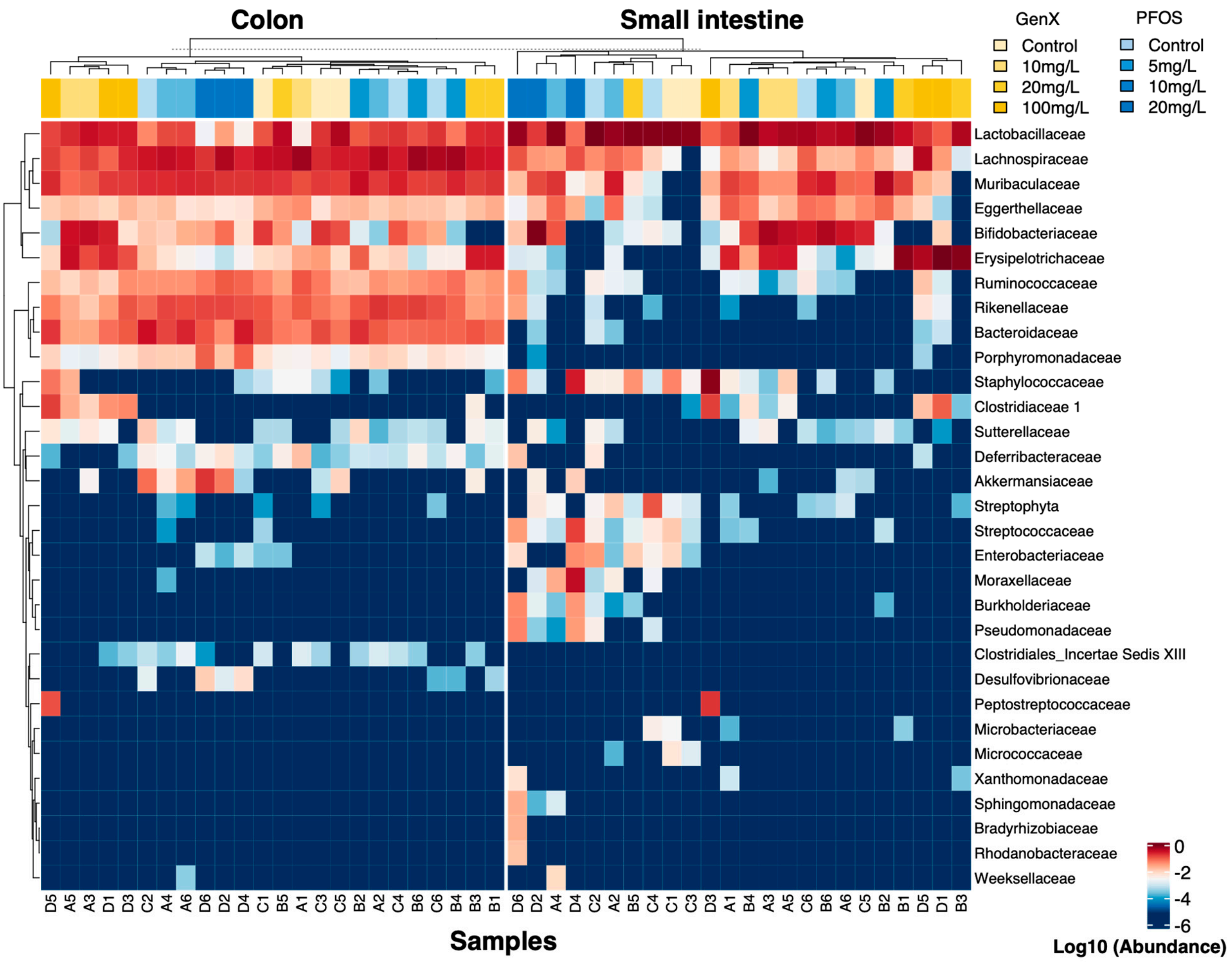

Figure S2. Heatmap of the taxonomic composition of all 16S samples on family level (according to rdp classification). The columns correspond to the samples; compound concentration in the diet is denoted with a top color bar. Figure shows the taxa with a total relative abundance of ≥ 0.5% across all samples. Hierarchical clustering was performed using the Euclidean metric and complete linkage.

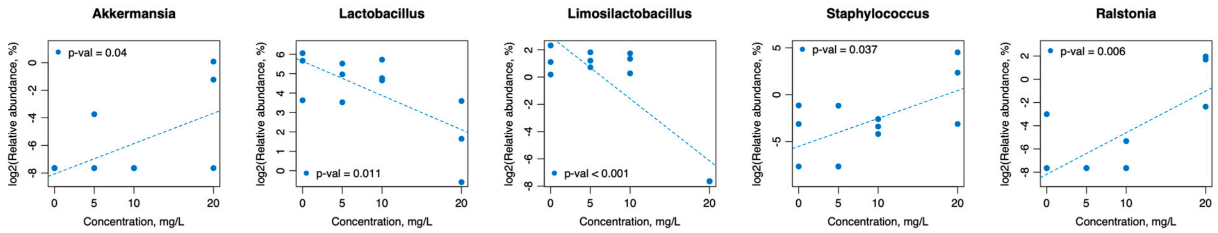

Figure S3. Changes in genus levels of 16S samples for small intestine and PFOS diets. Shown are genera with >80% confidence in rdp classification, >0.5% total abundance across all small intestine samples, and significantly non-zero slope of linear regression.

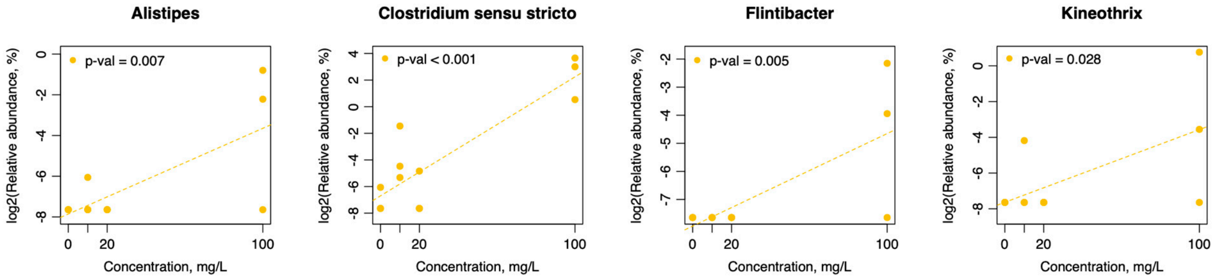

Figure S4. Changes in genus levels of 16S samples for small intestine and GenX diets. Shown are genera with >80% confidence in rdp classification, >0.5% total abundance across all small intestine samples, and significantly non-zero slope of linear regression.

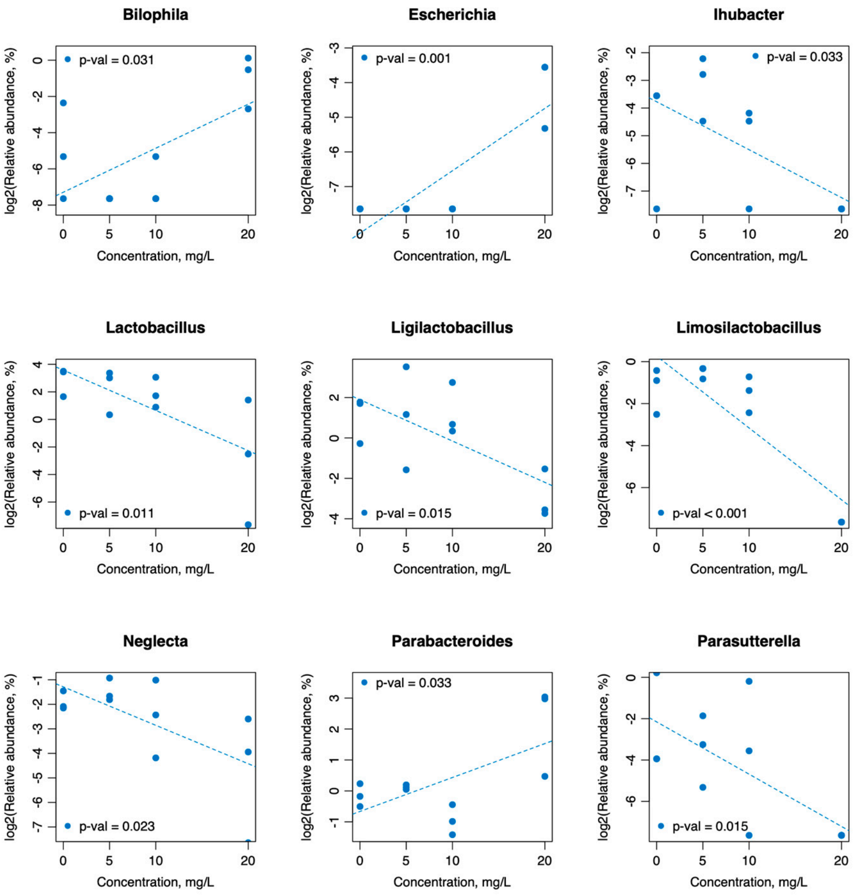

Figure S5. Changes in genus levels of 16S samples for colon and PFOS diets. Shown are genera with >80% confidence in rdp classification, >0.5% total abundance across all colon samples, and non-zero slope in linear regression.

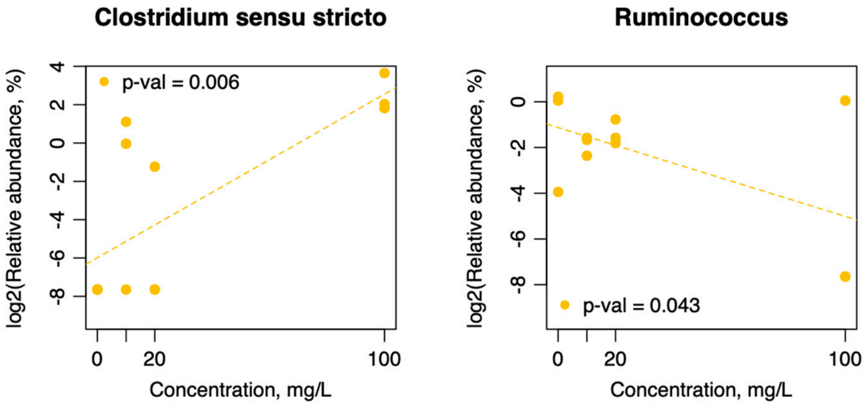

Figure S6. Changes in genus levels of 16S samples for colon and GenX diets. Shown are genera with >80% confidence in rdp classification, >0.5% total abundance across all colon samples, and significantly non-zero slope of linear regression.

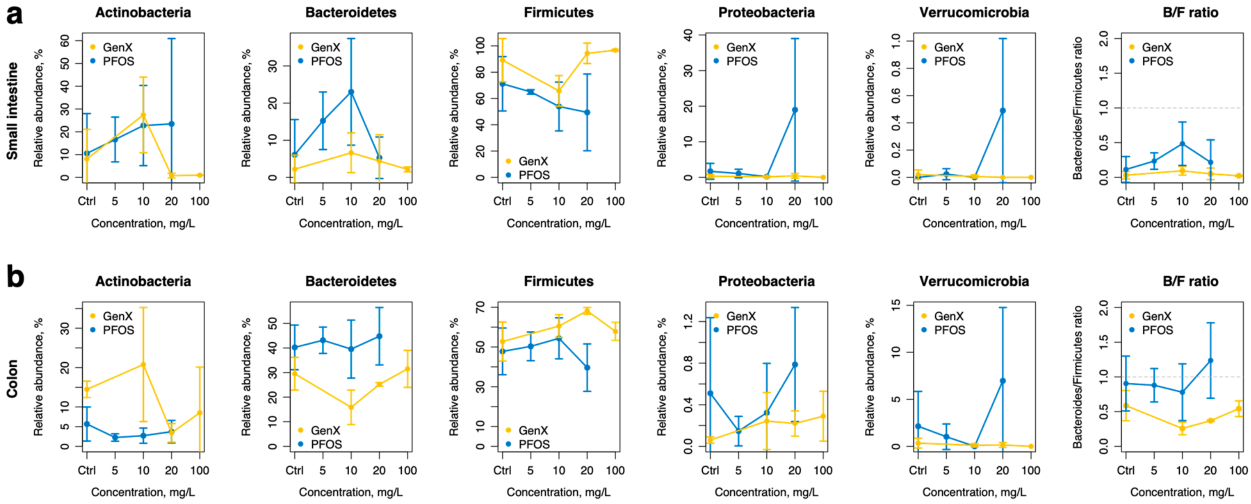

Figure S7. Changes in phylum levels of 16S samples for the small intestine (a) and colon (b) (>80% confidence in rdp classification). No significant changes were noted in these.

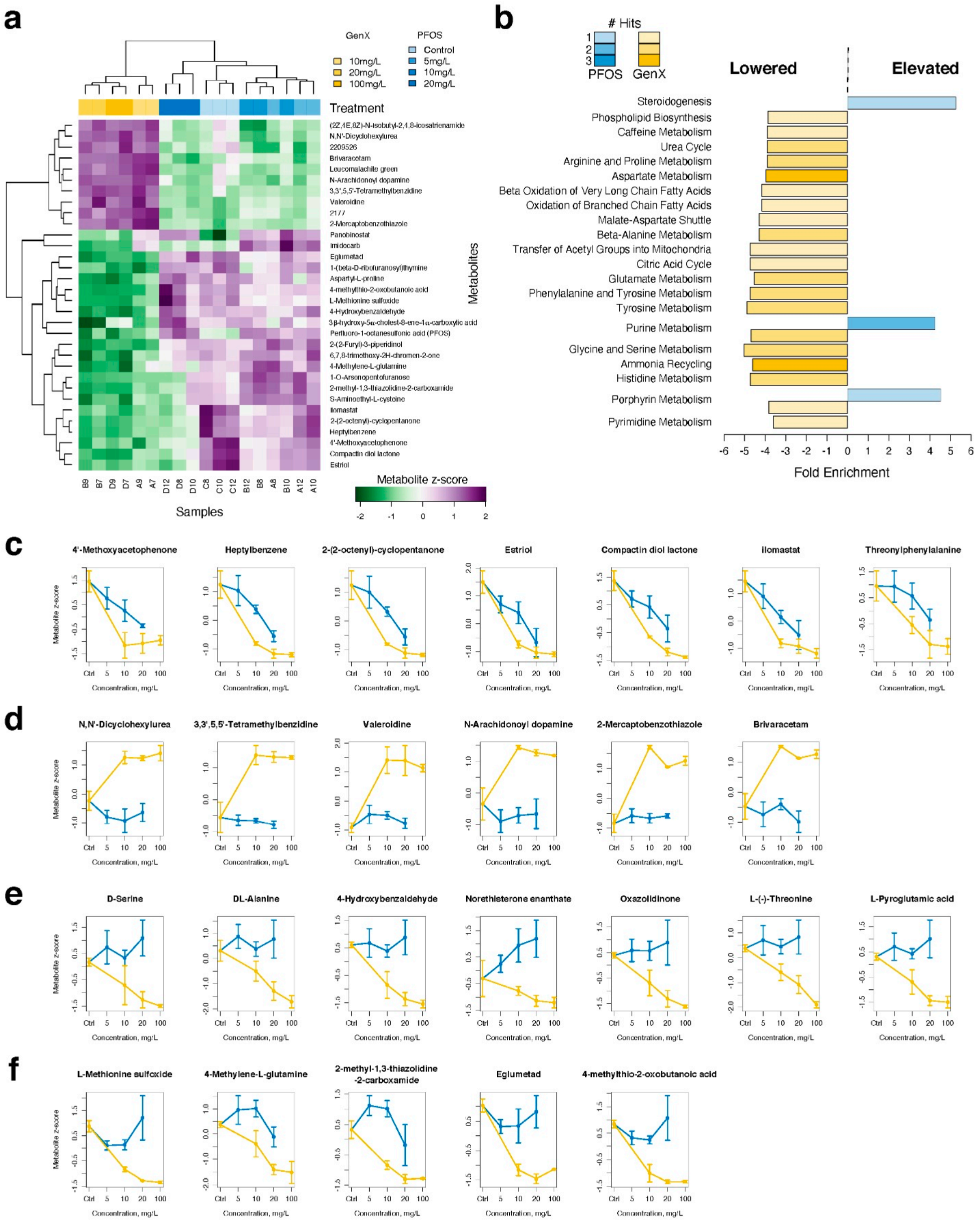

Figure S8. Small intestine metabolites significantly vary between different diets. (a) Heatmap of metabolites selected by ANOVA test (p-value<0.0001), peaks with low interquartile variability were filtered out before this analysis. Only metabolites with assigned potential chemical structures are shown (38/383). Color represents z-score value of log10 normalized metabolite concentrations. (b) Upregulated and downregulated pathways based on metabolite enrichment analysis in the small intestine. Only metabolites that have a significant association with the concentration of the toxic compounds (p-value of linear regression model < 0.05) were used for this analysis. Blue bars indicate pathways enriched in PFOS diets, red - in GenX, color intensity shows the number of metabolites present in the enriched/lowered metabolite set that belong to a given pathway. Pathway fold enrichment is calculated by the QEA analysis. Only those with a non-zero influence score or multiple hits and FDR < 0.05 are shown. Negative fold enrichment values indicate a set of pathways containing downregulated metabolites, positive - elevated ones. (c) Metabolites that systematically decrease with the concentration of toxic compounds. (d) Metabolites that are systematically elevated in GenX samples, but constant in PFOS. (e) Metabolites are constant in PFOS but systematically decreased in GenX. (f) Metabolites with non-linear response to the concentration of toxic compounds.

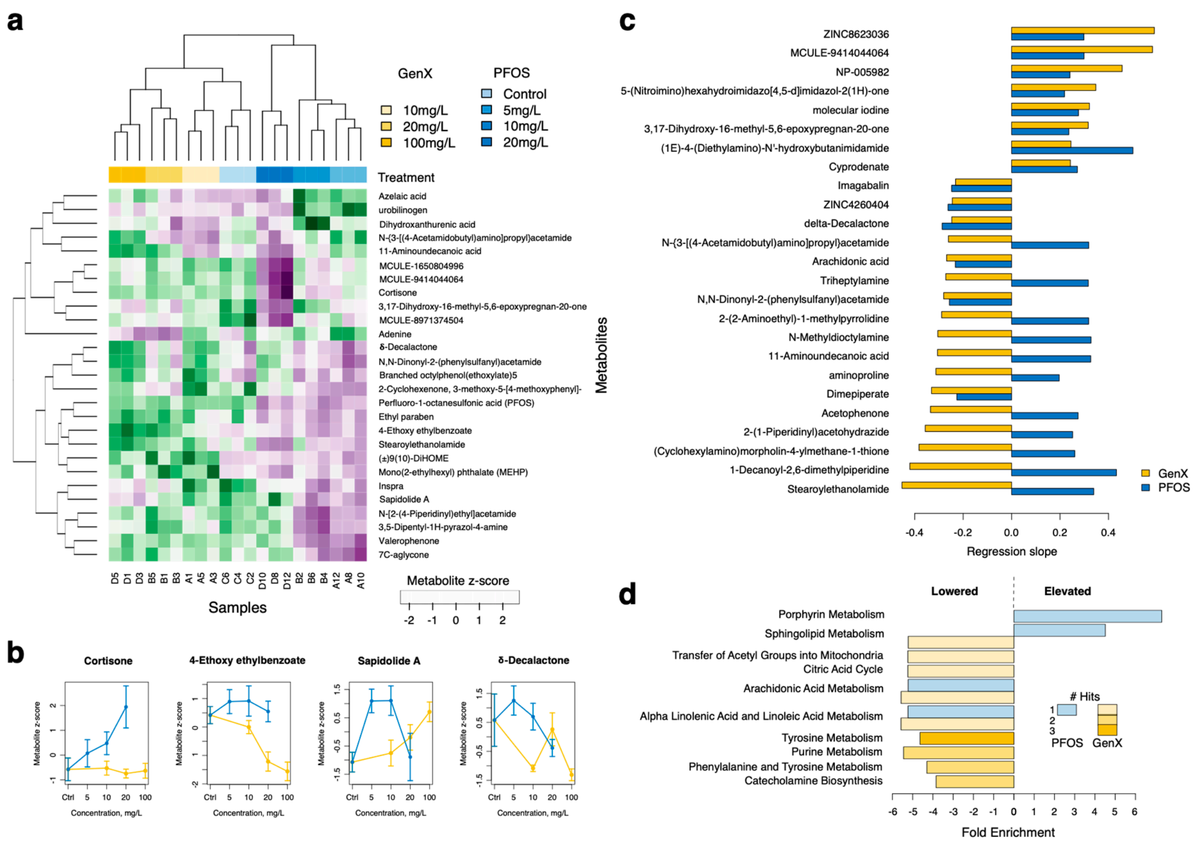

Figure S9. Colon metabolites significantly vary between different diets. (a) Heatmap of metabolites selected by ANOVA test (p-value<0.01), peaks with low interquartile variability were filtered out before this analysis. Only metabolites with assigned potential chemical structures are shown (36/137). Color represents z-score value of log10 normalized metabolite concentrations. (b) Selected metabolites that show some systematic trends with the concentration of toxic compounds. (c) Metabolites that were significantly elevated/lowered both in PFOS and GenX diets (p-value of linear regression model < 0.05). (d) Upregulated and downregulated pathways based on metabolite enrichment analysis in colon. Only metabolites that have a significant association with the concentration of toxic compounds (p-value of linear regression model < 0.05) were used for this analysis. Blue bars indicate pathways enriched in PFOS diets, red denotes GenX, color intensity shows the number of metabolites present in the enriched/lowered metabolite set that belong to a given pathway. Pathway fold enrichment is calculated by the QEA analysis. Only ones with a non-zero influence score or multiple hits and FDR < 0.05 are shown. Negative fold enrichment values indicate a set of pathways containing downregulated metabolites, positive - elevated ones.

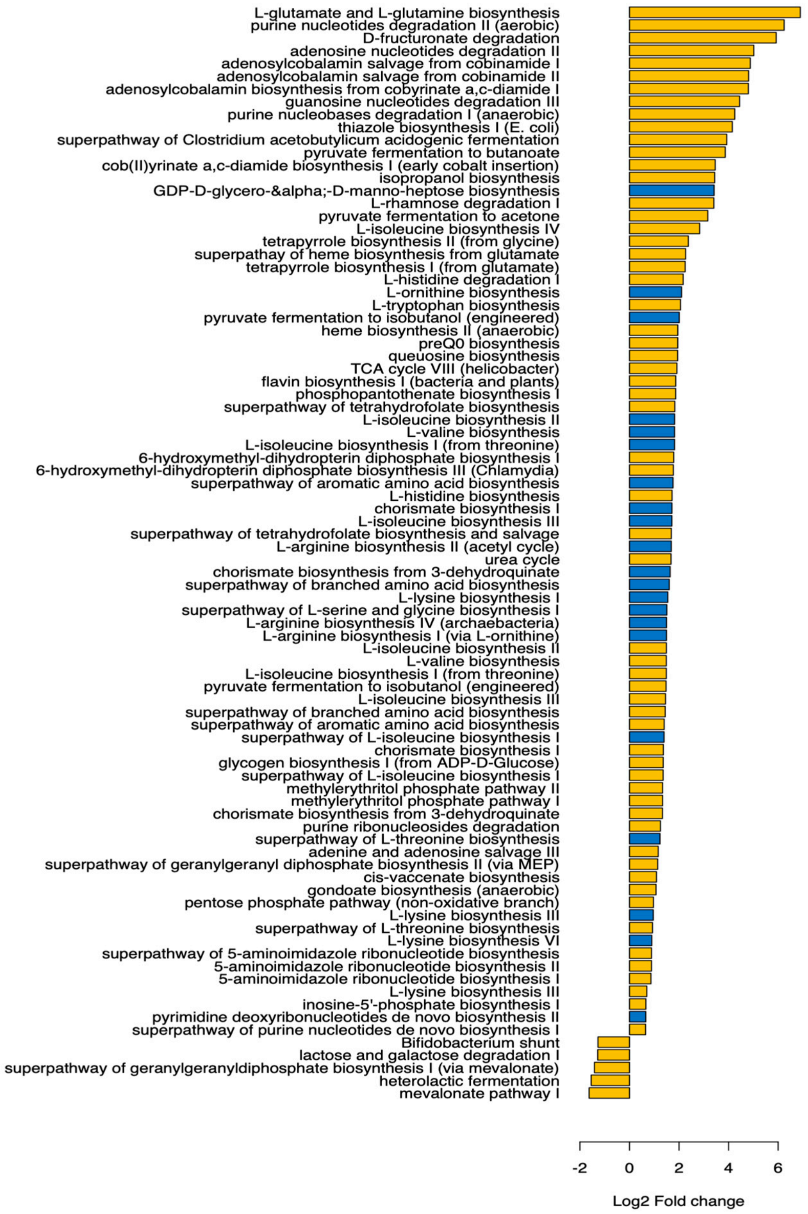

Figure S10. Differentially abundant pathways in the small intestine microbiome. We used PICRUSt2 predicted pathway abundances and linear regression model to identify pathways that are significantly enriched in PFOS (blue) / GenX (red) diets (p-value < 0.01). Only pathways with |Log2 Fold change|>1 are shown.

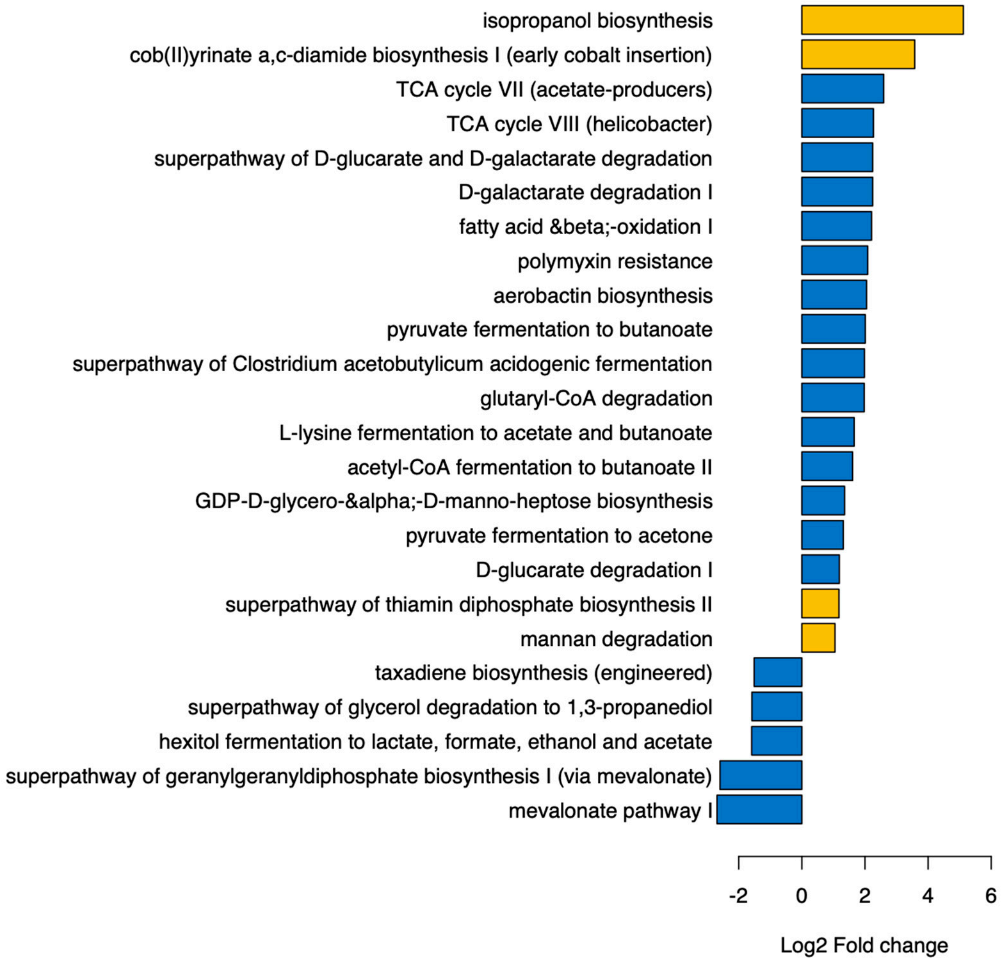

Figure S11. Differentially abundant pathways in the colon microbiome. We used PICRUSt2 predicted pathway abundances and linear regression model to identify pathways that are significantly enriched in PFOS (blue) / GenX (red) diets (p-value < 0.01). Only pathways with |Log2 Fold change|>1 are shown.

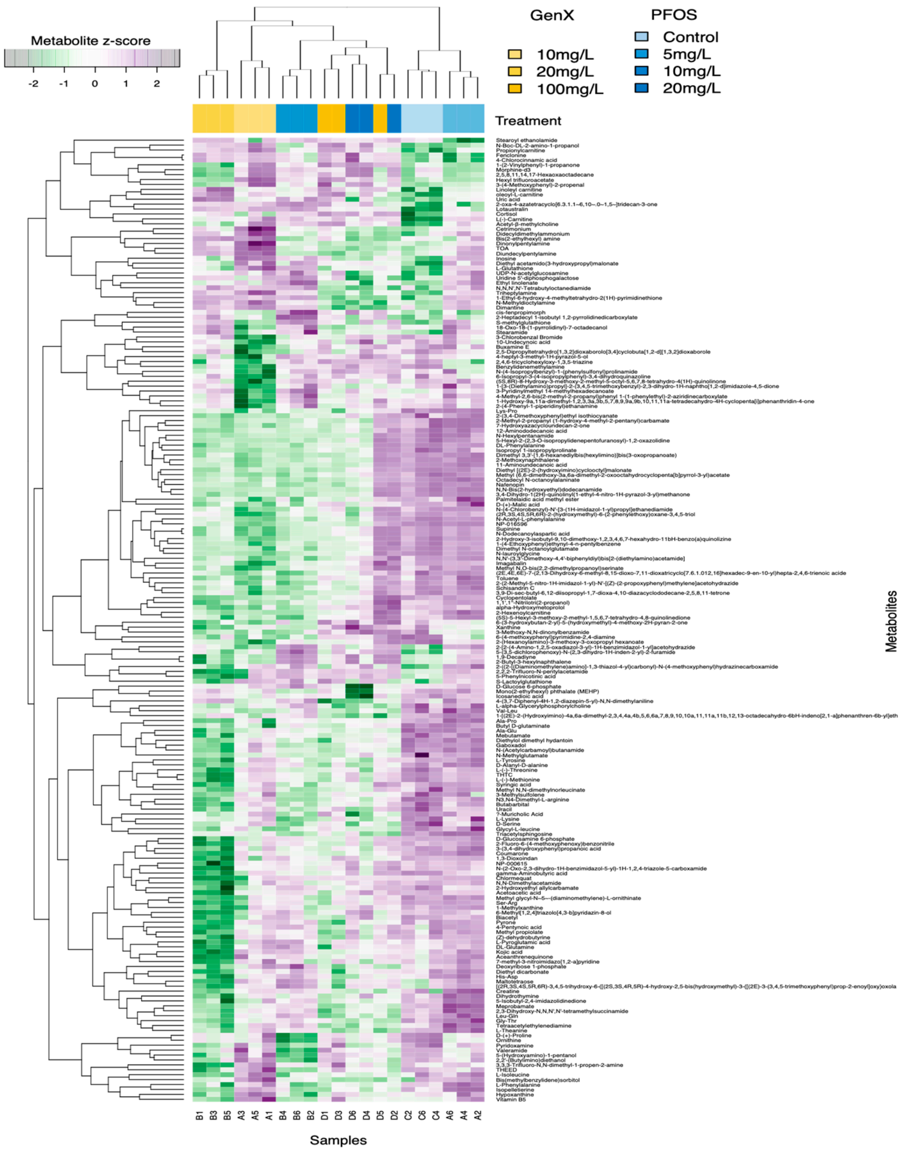

Figure S12. Heatmap of Liver metabolites which are significantly varying between different diets. Metabolites were selected based on the ANOVA test (FDR adjusted p-value < 0.01), peaks with low interquartile variability were filtered out before this analysis. Only 196 metabolites with names are shown (out of 491). Color represents z-score value of log10 normalized metabolite concentrations.
